# Epidemiology, molecular prevalence and prevention on canine parvovirus in India: A review

**DOI:** 10.6026/973206300200536

**Published:** 2024-05-31

**Authors:** Vanjavaka Pavana Jyothi, Mohana Subramanian Bhaskaran, Vijay A.K.B. Gundi

**Affiliations:** 1 MBIG Research Laboratory, Department of Biotechnology, Vikrama Simhapuri University, Nellore - 524 324, Andhra Pradesh, India; 2 Cisgen Biotech Discoveries, IITM Research Park, Chennai-600113, Tamilnadu, India

**Keywords:** Canine parvovirus, Haemorrhagic enteritis, vaccination, immunisation failures, maternal antibodies, CPV variants

## Abstract

Canine parvovirus (CPV) is a highly contagious and lethal virus that causes severe gastroenteritis and myocarditis in young dogs. In 1978, CPV has rapidly spread worldwide, resulting in outbreaks and high morbidity rates among dog populations. Over a decade, CPV has undergone genetic changes, leading to the emergence of different genotypes (CPV-2a, CPV-2b, and CPV-2c), which have expanded its host range to include cats and tissue culture cells. This review focuses on CPV-2 outbreaks in India from 2010 to 2023, analyzing gene lengths covering 274-438 amino acids in the VP2 gene which are collected from the NCBI database to investigate CPV epidemiology and diversity. The study highlighted substantial differences in seroprevalence over the period for CPV-2 (7%), CPV-2a (45%), CPV-2b (12%), and CPV-2c (36%). Our study found significant seroprevalence differences among CPV variants, with CPV-2a being the most prevalent, underscoring the need for effective diagnostic and preventive strategies.

## Background:

The etiological agent of canine parvovirus is one of dogs' most important viral diseases[1]. In the late 1960s, a minute canine virus (CPV1) was discovered gastrointestinal and respiratory infections in dogs [2]. All the symptoms are similar to FPV; therefore, it is believed that CPV originated as a host range variant of feline panleukopenia virus (FPV) or an adaptation to the new host dog via non-domestic carnivores such as minks and foxes. CPV is believed to have originated in the 1970s, and since then, genetic drift in the USA during the 1980s has resulted in two more variants - CPV-2a and CPV-2b [3]. A third variant, CPV-2c, was recognized more recently, in early 2000. The CPV-2c variant was first discovered in Vietnam in 2004 and has since rapidly spread worldwide [4]. In India, the disease is particularly rampant, and even vaccinated dogs are at risk of substantial illness and death. The virus spreads from dog to dog through direct or indirect contact with infected feces, making it a severe concern for pet owners everywhere. During the spring and early summer months, the Veterinary Hospital's emergency and urgent care department treats an average of two to four cases per week of puppies suffering from parvoviral enteritis, also known as parvovirus. This highly contagious virus attacks rapidly dividing cells in a dog's body, leading to severe symptoms such as vomiting, diarrhoea, weight loss, and dehydration. The virus can also attack white blood cells, leading to a significant decrease in blood cell count [5], and it leads to death. Sometimes, the weather gets warmer, and more unvaccinated dogs are introduced to public places like dog parks; the risk of exposure and transmission of the virus increases. Parvovirus is a resilient virus that can survive in a contained environment for up to eight months, making it easy to transmit among dogs [6]. To avoid exposure to infection, dogs need vaccination and prevention measures.

Controlling and preventing the spread of canine parvovirus (CPV) is difficult due to new variants affecting both domestic felines and wild carnivores. Accurate diagnosis can be aided by various serological and molecular tests, with live attenuated and inactivated vaccines being available. Although there is ongoing debate about the role of CPV variants in immunization failures, efforts towards eradicating CPV are recommended, particularly in countries where other canine viruses are less prevalent due to extensive vaccination. Mass vaccinations and strict disinfection protocols can help control the spread of the disease in stray, pet, and wild canine populations [7]. Although inpatient therapy is standard, outpatient protocols have shown a survival rate of over 80%. Maternal immunity interference is the primary cause of CPV vaccination failure. Traditional diagnostic methods have limitations, which has led to the development of molecular approaches for more sensitive and rapid detection. Continuous surveillance is necessary to assess the need for vaccine and diagnostic updates. In summary, parvovirus is a serious and potentially deadly disease that all dog owners should take seriously when symptoms start/immediately after they are sick. Early detection and treatment are crucial to increasing the chances of survival.

A thorough analysis is conducted on the various outbreaks and the extensive array of genetic variants observed within the Indian context from 2010 to 2023. The outbreak data was downloaded from NCBI, and a detailed analysis was done on epidemiological patterns, transmission dynamics, and the genetic diversity of the circulating pathogens. Furthermore, it meticulously explores the molecular changes exhibited by these variants, shedding light on their evolutionary trajectories and potential implications for public health. Through a meticulous examination of the available data, the review aims to offer valuable insights into the dynamics of infectious disease spread and evolution within the Indian subcontinent during this specific timeframe.

## Aetiology:

The International Committee on Taxonomy of Viruses classifies CPV-2 as Carnivore proto parvovirus 1, along with Feline panleukopenia virus (FPV), Mink enteritis virus (MEV), and Raccoon parvovirus (RPV) [8]. To distinguish it from CPV-1, the initial viral strain was named CPV-2. Until 1992, the canine minute virus (CAV-1) was assumed to be non-pathogenic. However, it is now understood to cause sickness in dogs [9]. The original strain of CPV-2, which is still included in most vaccines, is no longer common in the field. Instead, three different variants are distributed in different proportions around the world. These variants seem to be co-circulating in continental Europe, with types 2a and 2b being the most prevalent in Portugal, France, and Belgium; types 2a and 2c being the most prevalent in Italy; type 2a being the most prevalent in Eastern Europe; and type 2c being the most prevalent in the Iberian Peninsula. In Germany, all three variants are distributed equally. North and South America have a high prevalence of CPV-2b/2c and CPV2a/2c, respectively. Meanwhile, in Asia and isolated islands such as the UK, Australia, and Japan, types 2a and 2b are the most dominant strains [10], [11]. Limited reports from India suggest that all three strains are co-circulating in the Northern region, and there is a higher frequency of CPV-2a and 2c in the Southern part of the continent in India [12].

## Genome organisation:

Parvovirus is a Linear, negative sense ssDNA of about 4 to 6 kb in size. Non-enveloped, shape in round, icosahedral symmetry, 18-26 nm in diameter. It contains two open reading frames (ORF), which encode two nonstructural (NS1 & NS2) and two structural (VP1&VP2) proteins [13]. The nonstructural proteins are responsible for virus replication. Structural proteins, majorly VP2 capsid protein, play a role in antigenicity and immunogenicity, and they also have a critical role in controlling the host range and tissue tropism of viruses [14]. The replication of the genome occurs via a rolling hairpin mechanism. The host proteins are responsible for transcribing the genome into mRNA, with the number of promoters for mRNA transcription varying depending on the virus. Alternative splicing enables the expression of both structural and non-structural proteins [15] [16]. The parvovirus capsid comprises 60 protein subunits, consisting of VP1 (5-6 copies) and VP2 (54-55 copies). VP1 and VP2 have a similar structure, and their coding regions overlap except for a unique 143 amino acid N-terminal region. VP1 and VP2 are formed through alternative splicing of viral messenger RNAs [17] [18] [19]. Host proteases cleave the VP2 protein near its N-terminus to form another structural protein, VP3[20] [21]. The capsid proteins consist of a core with an eight-stranded anti-parallel β barrel that is highly conserved. The surface of the capsid has flexible loops between the β-strands that interact to form most of the surface. There is a raised region (spike) that is 22 Å long on the threefold axes, a deep depression (canyon) that is 15 Å deep, surrounding cylindrical structures at the fivefold axes, and a deep depression (dimple) that is 15 Å deep at the twofold axes. The threefold axes are the most antigenic region of the capsid and serve as a target for neutralizing antibodies [14] [20].

## Epidemiology:

Outbreaks of Parvovirus occur each year, with varying intensity and duration. This pattern is influenced by several factors, including the virus's antigenic properties, transmission capabilities, host range, and the community's susceptibility. The population's susceptibility is crucial in determining the severity of outbreaks and their impact on mortality and morbidity rates. Parvovirus has a unique ability to undergo changes in the antigenic characteristics of its capsid protein, which may result in significant antigenic shifts or minor antigenic drifts. Epidemics and pandemics are associated with antigenic shifts, while antigenic drifts cause localized outbreaks [19]. Young puppies are most susceptible to Parvovirus, with high mortality rates reported among adult dogs. The risk of morbidity and mortality is also raised in individuals with specific high-risk medical conditions, such as enteritis diseases, myocarditis, and extreme age. Recent data has demonstrated a heightened risk of complications for puppies at three weeks of age, and the virus is more infectious in males than females [22]. Furthermore, previous pandemics and seasonal outbreaks suggest that puppies may be at a higher risk for parvovirus complications than adult dogs.

An Australian surveillance study found a relationship between clusters of parvoviral enteritis and areas with lower socioeconomic status [23]. Various viral pathogens have been associated with diarrhea and enteritis in dogs, including canine distemper virus (CDV), canine enteric coronavirus, rotaviruses, astroviruses, adenoviruses, caliciviruses, and novel viruses like norovirus, kobuvirus, sapovirus, and possibly circovirus as well [24]. Canine enteric coronavirus mainly causes mild diarrhea in young puppies under six weeks old, and it could be present alongside other viral causes of gastroenteritis, such as CPV-2 variants. Although it has been identified as a rare cause of diarrhea in young dogs, it is not typically a significant cause. Here, we discussed canine parvovirus enteritis, the most widely recognized and pathogenic viral enteritis among dogs [11].

## Pathogenesis:

The canine parvovirus is a highly infectious virus that spreads through the ingestion or intake into the skin or oral mucosa of even a minimal amount of the virus. Upon entry, it promptly targets tissue in the intestine and lymphoid organs, where it begins to replicate. Subsequently, it disseminates throughout the body's tissues. The incubation period typically spans from 3 to 7 days. Upon entering the body, the virus rapidly multiplies the lymphatic system. During this replicative phase, it enters the bloodstream, leading to viremia. Within a few days, it reaches organs characterized by high cellular proliferation rates and continues replicating. The canine parvovirus infiltrates the hemopoietic system within the bone marrow, disrupting leukocyte production [25]. Consequently, the infected dog experiences a weakened immune response due to leukocytopenia. Additionally, the virus compromises the vascular barrier. It induces continual cellular sloughing within the intestinal villi, resulting in bloody diarrhea and heightening the risk of secondary bacterial infections as intestinal bacteria enter the bloodstream. Most fatalities associated with this virus stem from persistent vomiting and diarrhea, which lead to severe dehydration followed by hypovolemic shock.

Significant abnormalities are observed in cases of parvoviral myocarditis, including an enlarged heart with noticeable dilation of both the left atrium and ventricle. Although lung collapse is not typical, white frothy fluid may be present in the trachea and bronchi. Pulmonary edema and passive congestion in the liver are often evident, accompanied by varying degrees of ascites and pleural effusion. The ventricular myocardium frequently displays visible white streaks, indicating the presence of a cellular infiltrate. Some puppies may succumb to chronic decompensating left-sided heart failure weeks or even months after the sudden death of some littermates due to acute myocarditis. These delayed deaths are often attributed to pulmonary hypertension and myocardial dilation with scarring [26].

## Clinical Features:

Many symptoms can be seen in infected dogs, but that may not be seen in all the cases of parvovirus infectious dogs. The first and foremost symptoms are fever, diarrhea, and vomiting. It is possible for some dogs without displaying any symptoms of parvovirus except for weight loss. In order to give your dog the best chance of survival, it is important to pay immediate attention to the first sign of parvo. The typical signs of parvo are fever, diarrhea, vomiting, lethargy, shivering, and a lack of appetite or thirst. It is crucial never to disregard watery or bloody diarrhea, as this can quickly lead to dehydration and further severe complications. In fact, the parvo has no cure, but the symptoms are treatable; if dog owners respond quickly, treatment must be initiated in time, and the prognosis will be excellent. The difference in the severity of symptoms could be linked to the level of exposure and whether your dog has built up any immunity from previous illnesses. Canine Parvovirus (CPV) infection often affects puppies, leading to a range of symptoms such as anorexia, depression, vomiting, diarrhea, fever, and severe dehydration. A common symptom is leukopenia, which occurs when the white blood cell count falls below 2000-3000 cells/µL. Puppies with CPV enteritis may succumb to dehydration and show significant lesions in the gastroenteric tract, mainly involving the duodenum and jejunum. Hemorrhagic gastroenteritis is common, resulting in a thickened intestinal wall, dark red or purple serosal surface, and dark material or hemorrhagic fluid. Enlarged and congested mesenteric lymph nodes and Peyer's patches are observed in affected puppies. Due to the virus, the lymphocyte count decreases while the number of neutrophils increases, making the body more susceptible to bacterial infections, which can cause respiratory distress and death? The amount of leukopenia is a prognostic factor, and death can occur as early as two days due to bacterial dissemination or intravascular coagulation. Mortality rates vary depending on age and immunological status [27].

CPV myocarditis is a sudden death in infected puppies, often preceded by gastroenteric and dyspnoea. When the virus infected a group of unsuspecting dogs, acute myocarditis was usually discovered during the initial global CPV epizootics; however, this type is now infrequently seen in the field. Actually, puppies younger than 3-4 weeks old are the only ones that can develop CPV-induced myocarditis because, at that age, the myocardial syncytium is actively reproducing and vulnerable to viral replication. The virus predisposes dogs to degenerative heart disease, leading to heart failure. The delayed infections develop myocardial fibrosis, with myocardial lesions including non-suppurative myocarditis, multifocal infiltration, and intranuclear inclusion bodies. Today, however, almost all puppies receive MDA from their mothers, which shields them from parvovirus infection during the first several weeks of life, as most bitches have been vaccinated (or exposed to the virus) and have built a strong immune reaction [6].

## Diagnosis:

The preliminary diagnosis of parvovirus is a foul smell and bloody diarrhea. The same sign can be seen for other diseases also. Hence, laboratory diagnosis is essential to confirm the CPV infection. There are many tests available for CPV diagnosis.

## Haemagglutination Test:

The haemagglutination test is a rapid and simple test to detect the canine parvovirus in the test sample and is inexpensive. Red blood cells from a rhesus monkey, porcine, or feline are required to form haemagglutination in the test [28] post incubation at 4°C. If CPV is present in the sample, the virus has the ability to attach to and agglutinate red blood, and it can form hemagglutination [29]. However, the more common approach for clinical diagnosis of CPV infection in dogs is to use diagnostic tests that detect CPV in feces, blood, or other clinical samples from infected dogs.

## Electron microscopy:

Parvovirus morphology is visible through electron microscopy. A 4% agar block (1 cm2) evenly spread on the surface is required to conduct the test. The block is then negatively stained with a collodion solution of 2% phosphotungstic acid at pH 7 for 1 minute and placed on a carbon-coated grid. The purified preparations reveal negatively stained particles with diameters ranging from 23 to 26 nm, the typical size range for parvoviruses [30].

## Isolation of CPV:

It is possible to isolate CPV from fecal samples using primary cells such as A72, CRFK (Crandell Feline Kidney), and MDCK (Madin-Darby Canine Kidney). These infected cells may exhibit cytopathic effects under certain conditions of limited nutrients and temperature (at 35-37°C) [31]. The resulting supernatant can then be used for molecular characterization and attenuated studies.

## ELISA:

ELISA is a highly sensitive assay that uses antigen-antibody reactions. Either monoclonal or polyclonal antibodies can be used as primary antibody. The double sandwich ELISA is a quick, straightforward, sensitive, and suitable test for diagnosing CPV antigen in canine feces. Nowadays, the ELISA test has become the most common test used for detecting parvovirus in puppies [32].

## Polymerase chain reaction:

Polymerase Chain Reaction (PCR) is primarily a technique used to amplify DNA, and it is a qualitative method. However, PCR can be employed in molecular characterization, including identifying specific virus mutations or other genetic material. PCR is a laboratory technique to amplify and generate copies of a particular sequence of DNA. It involves a cyclic denaturation process, annealing, and extension carried out by a DNA polymerase enzyme. The result is an exponential increase in the number of copies of the target DNA [33] [34]. The subsequent analysis of PCR products on an agarose gel provides a qualitative assessment of the success of the amplification, and the resulting bands can be further analyzed or extracted for downstream applications, such as DNA sequencing [35]. The real-time-based technique is based on monitoring the amplification process and is used for gene expression analysis, viral load determination, and quantitative analysis. It can detect low titers of CPV in dogs" feces, which can help prevent CPV infection by adopting appropriate prophylactic measures, especially in kennels and shelters where this virus can cause severe outbreaks. Real-time PCR assay can identify dogs shedding CPV at low titers in their feces, helping to prevent CPV infection in kennels and shelters. TaqMan and SYBR Green-based PCR assays have been used to detect and quantify CPV-2 variants in fecal samples. MGB probes are an attractive tool for revealing single nucleotide polymorphisms in the capsid protein gene between CPV types [36].

## Nucleic acid sequencing:

Sequencing the PCR product, either as is or after cloning in a suitable vector, can be achieved with the aid of an automated DNA sequencer using an appropriate primer to type CPV strains. Following sequence analysis with suitable software, this technique is crucial in determining the exact variant of CPV present in a field sample. The nucleotide and amino acid sequence data can also be used to determine the percentage of homology and perform phylogenetic analyses of CPV-2 isolates from various regions. Based on the sequence analysis, it is possible to differentiate between CPV-2a and CPV-2b types, with none of the isolates being of the original CPV-2 type [37] [36]. In a subsequent study, both field isolates and vaccine strains of CPV were sequenced, revealing that vaccine strains were of CPV-2 type while field isolates were of CPV-2b type. CPV-2c variants have been identified in several countries through nucleotide sequence analysis.

## Mini Sequencing:

The mini-sequencing method determines specific sequences in a DNA sample using a limited number of nucleotides and a labeled primer. This method is based on single nucleotide polymorphism and is used to type CPV, including all four types (CPV2, 2a, 2b, and 2c) and feline panleukopenia virus. To differentiate between viral types, specific radio-labeled primers and conditions for the mini-sequencing reaction are designed [38] [39]. The protocol can be extensively implemented for diagnosing and differentiating CPV types in all veterinary hospitals and is also useful for epidemiological studies. Using this method, fecal samples can be analysed to detect the specific type of strain present in them, which can be helpful information in identifying the potential outbreaks of diseases caused by those strains and epidemiological studies. By identifying the strain, targeted vaccinations can be given to the surrounding areas, which can help prevent the spread of the disease. This process can significantly improve the efficiency of disease control measures and can help in the early detection and prevention of potential outbreaks.

## Geographical distribution of CPV Variants:

CPV-2 was first identified in dogs in 1978 [40] [41] and was named CPV-2 to differentiate it from the distantly related minute virus of canine (MVC), also known as CPV type 1 (CPV-1). CPV is believed to have originated from feline panleukopenia virus (FPV) as a host range variant. It has emerged through a direct mutation from FPV or adaptation to a new host (dogs) via non-domestic carnivores such as mink and foxes. That causes gastroenteritis and myocarditis in dogs. The genetic difference between CPV-2 and FPV is approximately 6-7 amino acids. Positive cases of CPV have been reported in various countries, such as the USA, Sweden, Italy, Germany, and Japan [42] [9] and later revealed that the original CPV-2 was replaced by a new antigenic variant, known as the "new" CPV, around 1980-1981. This new variant was labeled as CPV-2a and became prevalent in the dog population of the United States [43]. In 1979 and 1980, monoclonal antibodies were used to identify a novel strain of canine parvovirus type 2, which was named CPV-2a. In the 1980s, the virus underwent another change, leading to the emergence of a distinct variant known as CPV-2b. These newer strains have replaced the original canine parvovirus type 2 and are prevalent among dogs worldwide. The prevalence of CPV-2a and CPV-2b varies across different countries, with CPV-2b being the primary antigenic type in Europe, the USA, South Africa [44], [45] [46], and Turkey [47], Italy [48] and other European nations have higher rates of canine parvovirus type 2a [49] [4]. A novel antigenic variation has been identified in European and Southern Asian canines [50] [51]. These canine parvovirus type 2 mutants, previously known as the Glu-426 mutant and now known as cannel parvovirus type 2c (CPV-2c), were also discovered in Italy and Vietnam in 2000, and their pathogenicity is being explored [50]. The observations indicate that the CPV-2c strain does not present significantly more significant threats to shelter or pet canines than other CPV strains. However, the differentiation of CPV-2c from CPV-2b or 2a isolates solely based on clinical signs is indeed challenging. CPV-2c's clinical manifestations resemble those caused by previously known strains, including mucoid or hemorrhagic diarrhea, leukopenia, and lymphopenia [52] [53]. While some reports suggest CPV-2c might lead to more symptoms and higher mortality rates in adult dogs compared to type 2a and 2b, other studies propose that CPV-2c-infected dogs experience milder disease and lower mortality rates. Further investigation is necessary to comprehend CPV-2c's pathogenicity fully; gathering additional data on its clinical outcomes is crucial for devising appropriate preventive and therapeutic strategies [54].

The data presented in Table 1 was gathered from the NCBI database spanning from 2010 to 2023 across diverse regions of India. It was meticulously analysed, focusing on the geographical distribution of three CPV variants, specifically concerning the 426 residue at the VP2 gene. CPV 2a emerges as the predominant variant, with 324 isolates, followed closely by CPV 2c, with 262 isolates, as mentioned in Table 1. CPV 2b trails with 84 isolates, while CPV 2 has 48 isolates, and FPV accounts for 27 isolates. Particularly, Tamilnadu and Telangana exhibited elevated prevalence rates across all CPV variants compared to CPV 2a and CPV 2b. Pondicherry stands out for its notably high prevalence of CPV 2a. Meanwhile, Mizoram demonstrates a significant presence of CPV 2c. CPV 2c displays a broad geographical distribution observed across numerous states. Although CPV 2a is widespread, it is particularly pronounced in Tamilnadu, Telangana, and Pondicherry. Conversely, CPV 2b, though less prevalent, is still identifiable in several states. FPV, comparatively less prevalent than CPV variants, demonstrates a more scattered distribution across states. This comprehensive analysis offers valuable insights into the distribution patterns of distinct CPV variants across diverse regions of India, providing crucial information for understanding the epidemiology of canine parvovirus. The distribution of CPV and its various strains in each year from 2010 to 2023 is given in Table 2. The whole data was analyzed based solely on information available in NCBI, and it may vary from results obtained through a field survey.

**Figure 1:**
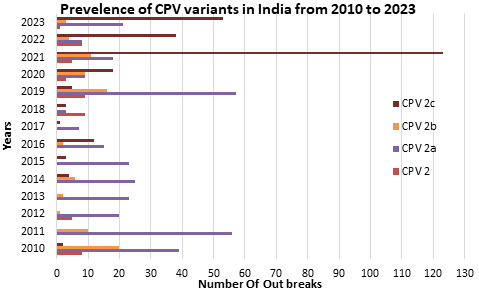
Graphical representation of the prevalence of CPV 2 outbreaks from 2010 to 2023

**Figure 2:**
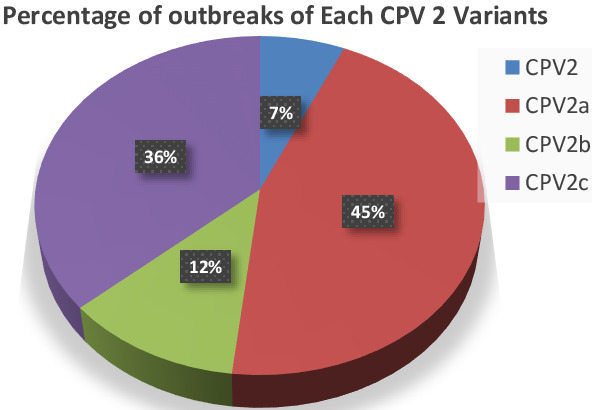
Percentage of strain distribution over the period of 2023

The graph outlines the yearly distribution of various strains of the canine parvovirus (CPV) from 2010 to 2023 in Figure 1. Each variant, including CPV 2, CPV 2a, CPV 2b, and CPV 2c, is represented across different years. CPV 2 displays fluctuating numbers, peaking in 2010, 2012, 2018, and 2023. CPV 2a, on the other hand, initially presented a high count in 2010 and exhibited varying numbers over subsequent years, with notable peaks in 2011, 2014, 2019, and 2020. CPV 2b's presence remains relatively consistent, with intermittent peaks observed in 2010, 2014, 2019, and 2020. In contrast, CPV 2c starts with minimal presence but steadily increases over time, with significant spikes in 2021 and 2022. The percentage of distribution is also mentioned in Figure 2. This comprehensive overview provides valuable insights into the temporal trends and fluctuations of different CPV variants over the specified period, contributing to a better understanding of the epidemiology of canine parvovirus based on the data available in the gene bank.

## Evolution of antigenic variants:

All variants of the CPV can be traced back to a common ancestor that emerged in the mid-1970s. This ancestor was closely related to the feline panleukopenia virus (FPV), a known virus infecting cats, minks, and raccoons. However, it does not infect dogs or cultured dog cells [55]. The sequences of the two viruses show an extremely high degree of similarity, with over 98% homology. The VP2 gene exhibits as few as six coding nucleotide differences, which occur at positions 3025, 3065, 3094, 3753, 4477, and 4498 [56]. CPV-2 was able to gain the ability to infect and replicate in canine hosts by a few genomic changes. However, it lost the capability to replicate in feline hosts. These changes were significant enough to alter the virus's host range and infectivity [57]. The CPV-specific antigenic epitope, responsible for the virus's canine host range, may be introduced by two changes at VP2 residues 93 from Lys to Asn and 323 from Asp to Asn between FPV and CPV [15]. Although CPV type 2 is closely related to FPV, it cannot infect cats. This limited host range is due to specific VP2 residues, specifically 80, 564, and 568, which are located near each other in the capsid structure [58].

Nucleotide changes were observed in CPV-2, but their significance is unknown. One year after its emergence in 1979, CPV type 2a, a variant of CPV, spread rapidly across the globe due to antigenic drift, replacing CPV type 2 strains. Compared to CPV type 2, CPV type 2a had five changes in the capsid sequence. These changes included VP2 residue substitutions of Met to Leu at position 87, Ala to Gly at position 300, and Asp to Tyr at position 305 [44]. CPV type 2a isolates were antigenically distinct from CPV type 2, and they infected and caused disease in cats. An antigenic variant of CPV type 2a (CPV type 2b) was recognized in 1984, and it differed in an antigenic epitope as a result of the substitution of VP2 at residue 426 from Asn to Asp and at residue 555 from Ile to Val [59]. These CPV-2a and CPV-2b are the predominant strains currently circulating in different dog populations and have completely replaced the original CPV-2 virus worldwide. Both the antigenic types coexist in various ratios in dog populations worldwide. Regaining the feline host range by CPV-2a and CPV-2b was likely to be a selective advantage of the virus [60]. In 2000, a new type of virus called CPV-2c was discovered in dogs in Italy. This virus differs from CPV-2b by just one amino acid at position 426, where Aspartic acid is replaced by Glutamic acid [61]. Since then, it has been reported in several other countries, including Vietnam, Spain, the United Kingdom, South America, North America, Portugal, and India [62]. At 426 positions, Glu mutation impacts the CPV-2 capsid major antigenic region located over the three-fold spike. Monoclonal antibodies were developed to detect various novel CPV-2 mutants [41]. Additionally, the CPV-2a isolates sequence revealed a reversion at position 555 to the sequence of FPV/CPV-2, Ile to Val. The impact of this mutation restricts the alterations among the antigenic variants CPV-2a, 2b, and 2c and the indistinguishable mutation at 426, which are Asn in CPV-2a, Asp in CPV-2b, and Glu in the CPV-2c.

## VP2 mutations reported in the antigenic variants:

**Figure 3:**
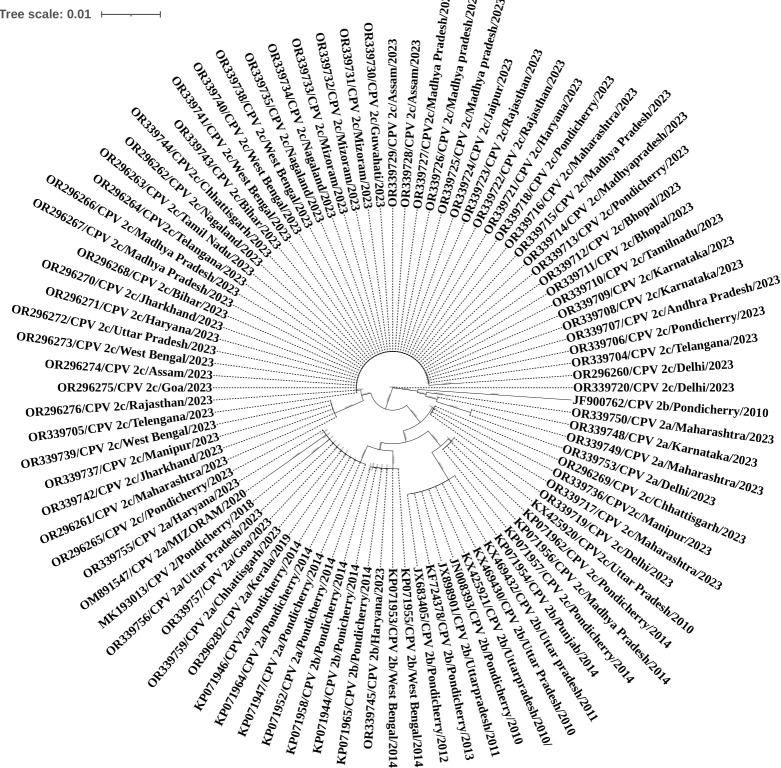
Phylogenetic trees were constructed based on the VP2 nucleotide and amino acid sequence types obtained in this study using all the CPV variants downloaded from the NCBI database.

The non-synonymous substitutions are also detected at the VP2 region in all variants. An amino acid change at VP2 position 297 (Ser to Ala) was observed both in CPV-2a and -2b. Which is located in a minor antigenic site close to epitope B, but no changes in the antigenicity of those variants have been reported. The identified mutation at 297 residues (Ser to Ala) has been designated as New CPV-2a and -2b [63]found in the USA, Asia, and Italy. The mutation at residue 440 in VP2 is located on top of the GH loop, which is the key antigenic site of the 3-fold spike. Zhou*et al.* also identified the mutation at position 440 (Alanine to Threonine) and at position 324 (Tryptophan to Isoleucine) in the capsid protein, which can affect the host range of CPV similarly to residue 323 [54] [64]. The mutations observed in the CPV-2a virus, specifically at positions 87, 101, 300, and 305, were likely acquired during the virus's evolution in raccoons. However, the changes at positions 300 and 305 were acquired when the virus returned to its canine host. The 300Asp mutation has recently been detected in domestic or wild felids in southern Asia and raccoons. This mutation is probably an adaptation of the virus to replicate better in the feline or raccoon hosts. The CPV-2a-specific residues at 87 and 101 positions were likely acquired during the evolution of the virus in raccoons, while the changes at 300 and 305 were acquired when the virus transferred back to the canine host. Additionally, 300Asp of CPV2a/2b has been detected in recent years in domestic or wild felids in southern Asia, as well as in raccoons. The mutation 300Asp is probably the expression of a further virus adaptation to replication in the feline or raccoon hosts [65] [66]. Although other wild animals, such as wolves, foxes, jackals, and coyotes, may also contributed to the spread and evolution of CPV-2, as they are susceptible to the disease [67] [68]. In this study, the phylogenetic analysis revealed that all the isolates were found to be phylogenetically closely related except a few isolates are New CPV-2a and 2b strains of India [69], and most of the sequences under this study were clustered together, showing distinct lineage showing in Figure 3.

The BLASTn tool was used to compare the DNA sequences of different CPV virus variants and determine their similarity level. The sequences were then translated into amino acid sequences using the Clustal software. Next, a phylogenetic tree was generated using the Mega 11 software and Itol software (https://itol.embl.de) to visualize the relationships between the CPV isolates obtained from NCBI. The Itol software is an online tool for the display, annotation, and management of phylogenetic and other trees. The maximum Neighbourhood joining tree method was used to depict the correlation between the VP2 gene partial nucleotide sequences of CPV isolates. The phylogenetic tree above was created using between 274 and 438 amino acids, covering the major antigenic sites prone to rapid mutation. A total of 91 sequences were analyzed, covering 426 amino acids. Among these was one sequence of CPV 2, 14 of CPV 2a, 13 of CPV 2b, and 61 of CPV 2c. The analysis revealed a significant incidence of CPV-2c in the present study, which indicates that all the variants are spread throughout India. Other than that, recent variants of new 2a and new 2b were also observed in minimal percentages. All the CPV isolates used in the present study had common amino acid substitutions except a few different position 297 SeráAla in VP2, indicating that the isolates were either new CPV-2a or new CPV-2b. Due to these jumping mutations and co-circulating, all variants fail the vaccinations. It is recommended that the vaccine manufacturers keep in mind that immunization must be protected against all strains.

## Recommendation for prevention or treatment perspective: 

Efforts towards CPV eradication are recommended, especially considering the reduced circulation of other canine viruses in extensively vaccinated countries. The role of canine parvovirus (CPV) variants in immunization failures is a subject of widespread debate [70]. Hence, the following is recommended:

1. Mass vaccinations and strict disinfection protocols can help control the spread of the disease in stray, pet, and wild canine populations.

2. A hospital stay is often necessary so that the dog can receive intravenous fluids and nutrients to replace the vast quantities lost via vomiting and diarrhea.

3. An intravenous drip is preferred because the digestive tract of stricken dogs is usually in distress and can't tolerate or absorb what the dog needs.

4. Blood transfusions may also be helpful to boost low blood cell counts that may result from CPV infecting the bone marrow.

5. Antibiotics may be an appropriate therapy for a dog suffering from CPV, administered either intravenously or as injections, to help fight the infection if intestinal bacteria have entered the bloodstream.

6. Effective vaccinations have significantly reduced the canine parvovirus (CPV) threat in dogs. Timely medical therapy also helps dogs recover and develop lifelong immunity against the infecting strain.

7. Vaccination should be initiated every 3 to 4 weeks from 6 to 16 weeks for puppies.

8. A booster vaccination is recommended one year later and then at three-year intervals afterward.

9. If an infected dog has contaminated your home and yard, disinfect them before getting a new pet.

10. Although CPV is relatively resistant to cleaning agents, bleach can clean or inactivate it in the surrounding area.

11. An appropriate way to sanitize all indoor areas, such as bedding, food and water bowls, and surfaces that previously contained an infected dog, is to use a mixture of one part bleach and around 30 parts water.

## Discussion:

The evolution of FPV to CPV and the re-emergence of CPV with different variants in India, which represents high mutational changes in the VP2 region, have been discussed in this report. Epidemiological studies in India have provided information about the distribution of three antigenic variants of CPV in the dog population over the past 13 years, from 2010 to 2023. These studies found that the original CPV-2 has disappeared in the dog population, and there is no difference among the antigenic variants in terms of pathogenicity. Sequences were downloaded from the NCBI in India to study the diversity of CPV isolates. A phylogenetic tree was constructed using partial VP2 gene sequences to estimate the viral phylogenetic relationships. The classification system based on single amino acids (297and426) of the VP2 protein does not reflect the phylogenetic relationships of the strains, better supported by the proposed "clade" or "lineage/sub-lineage" new classification criteria. A total of 91 sequences were used for the phylogenetic tree covering all the variants (CPV 2, CPV 2a, CPV 2b, and CPV 2c). However, the above study reveals CPV-2b variant has a lower prevalence than CPV-2a and CPV-2c. Battilani*et al.* found that CPV-2b was more genetically stable than CPV-2a, as its sequence analysis showed the highest fraction of non-synonymous mutations, highlighting the significant phenotypic effects of the accumulated mutations over time. An increased prevalence of CPV-2a was also reported in our study from the West India region. As also observed in this study, phylogeny clustering is based on the single VP2 amino acid residue 426 (CPV-2a/2b/2c) and the geographic origin and sample collection period. Therefore, a more comprehensive evolutionary analysis further supports the hypothesis to consider the CPV antigenic variants as variants of CPV-2a rather than distinct subtypes and could be considered a more reliable tool in outbreak tracing.

## Conclusion:

Canine parvovirus is a dangerous, highly contagious virus that infects young dogs and is difficult to eliminate from homes or kennels due to its survival outside a host. Modified live vaccines can be safe if proper protocols are followed, but there is a gap in vaccine coverage for puppies due to maternal antibodies, and new strains may emerge as the virus mutates. Developing homologous vaccines with nanoparticle delivery mechanisms may be necessary to combat new strains like CPV-2c. Adequate treatment is necessary for infected dogs involving fluid replenishing and symptom management can improve patient survivability. Extensive research is needed to understand the prevalence of different CPV strains and prevent outbreaks. Ongoing surveillance is essential for enhancing our understanding of CPV dynamics and ensuring the development of updated vaccine strains that are ready for future challenges.

## Figures and Tables

**Table 1:** The data collected from NCBI shows the geographical distribution of CPV outbreaks in India.

**Name of State**	**FPV**	**CPV 2**	**CPV 2a**	**CPV 2b**	**CPV 2c**
Andhra Pradesh			5		7
Assam			1		4
Arunachala Pradesh			4		5
Bihar					2
Jharkhand					2
Goa	1		7		1
Tamilnadu	20	1	38	5	3
Telangana	1	1	38	25	6
Pondicherry	1	15	81	13	8
Kerala	2		7		
Karnataka	1		1		2
Mizoram	1	7	11	4	38
Gujarat		6	1	1	2
Uttar Pradesh		14	65	12	13
Chhattisgarh			1	4	5
Tripura			4		28
Haryana			15	6	1
Delhi		1	2		4
Maharashtra			21	10	31
Madhya Pradesh		2			12
Manipur					24
Odisha			2		
Nagaland			2		20
Rajasthan					3
Sikkim			1		11
Punjab			2		
Meghalaya			15		18
Himachalapradesh				1	
Uttarakhand				1	1
West Bengal				2	11
Total no. of isolates	27	48	324	84	262

**Table 2:** List of the number of different variants of CPV-2 for each year from 2010 to 2023

**Year Virus**	**2010**	**2011**	**2012**	**2013**	**2014**	**2015**	**2016**	**2017**	**2018**	**2019**	**2020**	**2021**	**2022**	**2023**
CPV 2	8	0	5	0	0	0	0	0	9	9	3	5	8	1
CPV 2a	39	56	20	23	25	23	15	7	3	57	9	18	8	21
CPV 2b	20	10	1	2	6	0	2	0	0	16	9	11	4	3
CPV 2c	2	0	0	0	4	3	12	1	3	5	18	123	38	53

**Table 3:** The evolutionary process based on mutations that occurred in the VP2 capsid protein of Parvovirus (The arrow symbol indicates amino acid change)

**Strain**	**Year**	**Amino acid changes at VP2**						
FPV	1900	Position	80	93	103	323	564	568
		Amino acid	K	K	V	D	N	A
CPV2	1974-1978	Amino acid	R	N	A	N	S	G
CPV2	1974-1978	Position	87	101	300	305	555	
		Amino acid	M	I	A	D	V	
CPV2a	1979	Amino acid	L	T	G	Y	I	
CPV 2a	1979	Position	426	555				
		Amino acid	N	I				
CPV 2b	1984	Amino acid	D	V				
CPV 2b	1984	Position	426					
		Amino acid	D					
CPV 2c	2000	Amino acid	E					
